# Impaired Functions of Macrophage from Cystic Fibrosis Patients: CD11b, TLR-5 Decrease and sCD14, Inflammatory Cytokines Increase

**DOI:** 10.1371/journal.pone.0075667

**Published:** 2013-09-30

**Authors:** Karin Simonin-Le Jeune, André Le Jeune, Stéphane Jouneau, Chantal Belleguic, Pierre-François Roux, Marie Jaguin, Marie-Thérèse Dimanche-Boitre, Valérie Lecureur, Caroline Leclercq, Benoît Desrues, Graziella Brinchault, Jean-Pierre Gangneux, Corinne Martin-Chouly

**Affiliations:** 1 Université de Rennes 1, Structure Fédérative de Recherche Biosit, F-35043 Rennes, France; 2 Institut de Recherche Santé Environnement & Travail (IRSET), Institut National de la Santé et de la Recherche Médicale (INSERM), U1085, team ‘Stress Membrane and Signaling’, F-35043 Rennes, France; 3 Equipe Microbiologie "Risques Infectieux" EA 1254, F-35043 Rennes, France; 4 Institut de Recherche Santé Environnement & Travail (IRSET), Institut National de la Santé et de la Recherche Médicale (INSERM), U1085, team ‘Chemical contaminant immunity and inflammation’, F-35043 Rennes, France; 5 Centre Hospitalier Universitaire de Rennes, Centre de Ressource et de Compétences de la Mucoviscidose, F-35064 Rennes, France; 6 Centre Hospitalier Universitaire de Rennes, Service de Parasitologie-Mycologie, F-35064 Rennes, France; INSERM, France

## Abstract

**Background:**

Early in life, cystic fibrosis (CF) patients are infected with microorganisms. The role of macrophages has largely been underestimated in literature, whereas the focus being mostly on neutrophils and epithelial cells. Macrophages may however play a significant role in the initiating stages of this disease, *via* an inability to act as a suppressor cell. Yet macrophage dysfunction may be the first step in cascade of events leading to chronic inflammation/infection in CF. Moreover, reports have suggested that CFTR contribute to altered inflammatory response in CF by modification of normal macrophage functions.

**Objectives:**

In order to highlight possible intrinsic macrophage defects due to impaired CFTR, we have studied inflammatory cytokines secretions, recognition of pathogens and phagocytosis in peripheral blood monocyte-derived macrophages from stable adult CF patients and healthy subjects (non-CF).

**Results:**

In CF macrophage supernatants, concentrations of sCD14, IL-1β, IL-6, TNF-α and IL-10 were strongly raised. Furthermore expression of CD11b and TLR-5 were sorely decreased on CF macrophages. Beside, no difference was observed for mCD14, CD16, CD64, TLR-4 and TLR1/TLR-2 expressions. Moreover, a strong inhibition of phagocytosis was observed for CF macrophages. Elsewhere CFTR inhibition in non-CF macrophages also led to alterations of phagocytosis function as well as CD11b expression.

**Conclusions:**

Altogether, these findings demonstrate excessive inflammation in CF macrophages, characterized by overproduction of sCD14 and inflammatory cytokines, with decreased expression of CD11b and TLR-5, and impaired phagocytosis. This leads to altered clearance of pathogens and non-resolution of infection by CF macrophages, thereby inducing an exaggerated pro-inflammatory response.

## Introduction

Lung problem is the dominant clinical feature consequence of chronic excessive inflammation, and accounts for morbidity and mortality in patients with Cystic Fibrosis (CF), an autosomal recessive disorder caused by mutations in gene encoding Cystic Fibrosis Transmembrane conductance Regulator (CFTR) protein [1,2]. Persistent inflammation is notably due to the dehydration of airway liquid leading to depletion of the periciliary layer and production of highly viscoelastic mucus, which significantly impacts mucociliary clearance [3]. Beside alteration of the mucociliary clearance system, CFTR mutations might affect other functions of bronchial epithelial cells including the internalization of *Pseudomonas aeruginosa* and the release of inflammatory mediators [4,5].

The role of macrophage has largely been overlooked in CF pathophysiology, the focus being mostly on neutrophils and epithelial cells. However macrophage dysregulation could impair resolution of inflammation *via* an inability to act as a suppressor cell, then leading to chronic inflammation/infection. Some studies have recently suggested that altered properties of immune CF cells may also contribute to the uncontrolled inflammation in CF lung. Studies on neutrophils have especially shown that CFTR, expressed on phagolysosomes membrane, is crucial for the chlorination reactions involved in bacterial killing by human neutrophils [6,7]. Researches using CFTR knockout (CFTR^-/-^) mice have demonstrated CFTR contribution in regulation of phagosomal pH in murine alveolar macrophages. Thereby CFTR-deficient macrophages failed to acidify lysosomes and phagolysosomal compartments and displayed an altered bactericidal activity [7]. Furthermore, in response to lipopolysaccharide (LPS) from *P. aeruginosa*, bronchoalveolar lavage fluids (BALF) of CFTR^-/-^ mice were characterized by significantly higher concentrations of pro-inflammatory cytokines released by macrophages, such as IL-1α, IL-6, G-CSF and IL-8, than BALF from wild type (WT) mice. Excessive production of these cytokines was also confirmed in bone marrow and alveolar macrophages from CFTR^-/-^ mice after *in vitro* stimulation with LPS from *P. aeruginosa* [8]. Regarding phagocytosis and microbicidal activity, a higher percentage of live bacteria was observed in monocyte-derived macrophages differentiated with M-CSF from CF patients infected with *P. aeruginosa* compared with macrophages from healthy subjects; although an overall reduction in live bacteria is observed both in cells from patients and healthy subjects [9]. Finally, CFTR defect and excessive inflammation in human and murine macrophages have been associated with an abnormal signaling and trafficking of TLR-4, the LPS receptor [10].

Although these studies support the hypothesis that alteration of macrophage functions induces chronic infections and hyper-inflammation, human macrophage pivotal role remains unknown in CF. Our work highlights excessive inflammation and the defect of macrophage functions during CF chronic infections.

## Methods

### CF patients

The experiments were conducted according to the Good Clinical Practice guidelines [11] and approved by the Ethical Committee of human subjects of the Rennes University Hospital (France, Ethics No. 11/38-827). All patients included in this study gave their written informed consent. Forty-six stable adult patients with CF were recruited at the ‘Centre de Ressources et de Compétences de la Mucoviscidose’ of the Rennes University Hospital (France). CF patients considered for inclusion were Caucasian, 26 males and 20 females, aged between 18-52 years (mean age: 30±1). The CF diagnosis was based on typical clinical manifestations of the disease and confirmed by positive sweat tests and by CFTR gene mutation detection. The stable patients were defined by the absence of change in their symptoms in the 3 months prior to the study. All the patients with CF had medication at the time of blood collection, including azithromycin (38%), aerosol of DNAse (62%), inhaled corticosteroids (60%) and azole therapy (40%). Patients who were selected were not on oral corticosteroids therapy at the time of blood collection, as this may have influenced inflammatory phenotypes. The clinical features of these patients are reported in Table S1. According to the Force Expiratory Volume in one second (FEV1) values (% predicted), the majority of our patients (30/46) had mild to moderate lung disease (respectively FEV1 values ≥55%). Twenty-three patients had microbiological evidence of *P. aeruginosa* colonization, eighteen of *Aspergillus fumigatus* and thirty-one of *Staphylococcus aureus*. CF genotype was representative of CF French population with 48% of F508del/F508del mutations, 41% of F508del/other mutations and 11% of no F508del mutations [12]. The blood monocytes count for CF patients was within normal range with a median number of 0.70x10^9^/L (range 0.34-1.27x10^9^/L).

### Cell cultures and treatments

Leukocytes were isolated by Ficoll gradient centrifugation, as previously described [13]. Peripheral blood mononuclear cells from non-CF healthy subjects (written consent to use of blood sample for research protocol according regulation for blood transfusion of French blood organization, EFS, Rennes), were seeded at 15x10^6^ leukocytes/mL whereas cells from CF patients were seeded at 8x10^6^±1x10^6^ leukocytes/mL, according to specific blood count of each patient. Monocytes, selected by 1-hour adhesion step, were differentiated for 6 days by GM-CSF (400 UI/ml, Genzyme Corporation, Cambridge, UK) in RPMI 1640 medium supplemented with 2 mM glutamine, antibiotics and 10% FCS (Lonza, Saint-Beauzire, France). Monocyte-derived macrophages characterization was assessed using flow cytometry by analyzing a specific membrane cluster of differentiation, CD71. Membrane expression of the macrophagic marker CD71 was almost four times higher in non-CF (mean MFI of 19.76±3.1, n=25, p<0.01) and CF macrophages (mean MFI of 14.11±1.41, n= 20, p<0.001) than in monocytes (mean MFI of 4.98±0.76, n=5) underscoring differentiation of monocytes into macrophages (Figure S1).

To study CFTR defect, macrophages were chronically (each day for 72 h) exposed to an inhibitor of CFTR function (CFTR_inh-172_, 10 µM, Sigma-Aldrich, Saint-Quentin-Fallavier, France).

### Flow cytometric immunolabelling assay

Phenotypic analysis of macrophages was performed using flow cytometric direct immunofluorescence as previously described [13]. Antibodies used in this study were provided in File S1. After gating for CD71^+^ cells, specific mean fluorescence intensity (MFI) for each protein was recorded (10,000 events). Thereafter, cells were analyzed with FC500 flow cytometer using CXP analysis software (Beckman Coulter, Villepinte, France). Results were expressed as MFI calculated as follows: mean fluorescence (mAb of interest)/mean fluorescence (isotypic control).

### Measurement of inflammatory cytokines, sCD14 and mCD14 concentrations

IL-1β, IL-8, **IL-6, TNF-α, IFN-γ, IL-10** and sCD14 levels from supernatants of macrophage cultures were quantified by ELISA according to the manufacturer’s instructions (R&D system Europe, Lille, France).

mCD14 levels were determined using a cell-based ELISA. Leukocytes (2x10^5^ cells per well) were seeded into 96-multiwell plates and after differentiation into macrophages, treatments were applied. Then, cells were fixed one night with 4% paraformaldehyde. CD14 ELISA was then realized as describe above for sCD14 (R&D system Europe).

### Phagocytosis assay

Phagocytosis capacity was evaluated by two different methods. The first used heat-inactivated *Escherichia coli* linked to fluorescein by the way of the ‘Vybrant Phagocytosis Assay kit’ (Molecular Probes, Life Technologies, Saint-Aubin, France) according to the manufacturer’s instructions described in File S1.

The second method was a microbiological assay with live *P. aeruginosa*. Macrophages were seeded in 24-well plates (2x10^5^ cells/well). *P. aeruginosa* (strain ATCC27853, LGC Standards, Molsheim, France) were grown in tryptic soy medium (Sigma-Aldrich) at mid exponential phase and suspended in serum-free RPMI 1640 medium after wash. Bacterial population was adjusted in order to infect macrophages at a multiplicity of infection (MOI) of 100. Infection occurred during 1 hour at 37°C with 5% CO_2_. Then, cells were gently washed with PBS and incubated for 1 hour with gentamycin (150 µg/ml, Sigma-Aldrich) to kill extracellular or adhered remaining bacteria. Intracellular/phagocytosed bacteria were released by lysing the cells with cold water containing 0.025% Triton X-100 (Sigma-Aldrich) and count by plating serial dilutions on appropriate agar medium. Results are expressed in 10^4^ CFU of *P. aeruginosa/*10^5^ macrophages.

### Western blot analysis

Membrane-bound proteins were extracted of macrophages by lysing the cells with hypotonic lysis buffer (10 mM Tris-HCl pH 7.5, 10 mM NaCl, 1 mM MgCl_2_, 4 mM PMSF, 10 mM NaF, 10 mM NaPPi, 1 mM Na _3_VO_4_ and proteases inhibitors cocktail Roche mini complete, Roche, Boulogne-Billancourt, France). Macrophages were incubated at 4°C for 30 minutes in lysis buffer before vortex for 30 seconds. The supernatant obtained by subsequent centrifugation at 5,000 g for 10 minutes at 4°C contained membrane-bound proteins. Proteins (30 µg) were then separated on 5% SDS-polyacrylamide gels, transferred to nitrocellulose membrane. Then, membrane was subjected to Western blotting using the following primary antibody: rabbit polyclonal anti-CFTR (1/200, Cell Signaling Technology, Ozyme, Saint-Quentin-en-Yvelines, France). Equal protein loading was confirmed using mouse monoclonal anti-HSC-70 (1/3000, Santa Cruz, Tebu-Bio, Le Perray-en-Yvelines, France). Horseradish peroxidase-conjugated goat anti-rabbit secondary antibody (Dako, Trappes, France) was used at 1/3500 and proteins detected using enhanced chemiluminescence.

### Statistical analysis

The number of subjects and experiments used in each group is stated in the respective figures. A non-parametric Mann-Whitney test was used to assess the statistical significance of differences between groups: p-value <0.05 was considered significant.

## Results

### CF macrophages present a pro-inflammatory phenotype

In order to resolve infection, macrophages secrete a wide array of powerful biological substances, including regulatory factors such as interleukins, which lead to inflammation.

**Figure 1:**
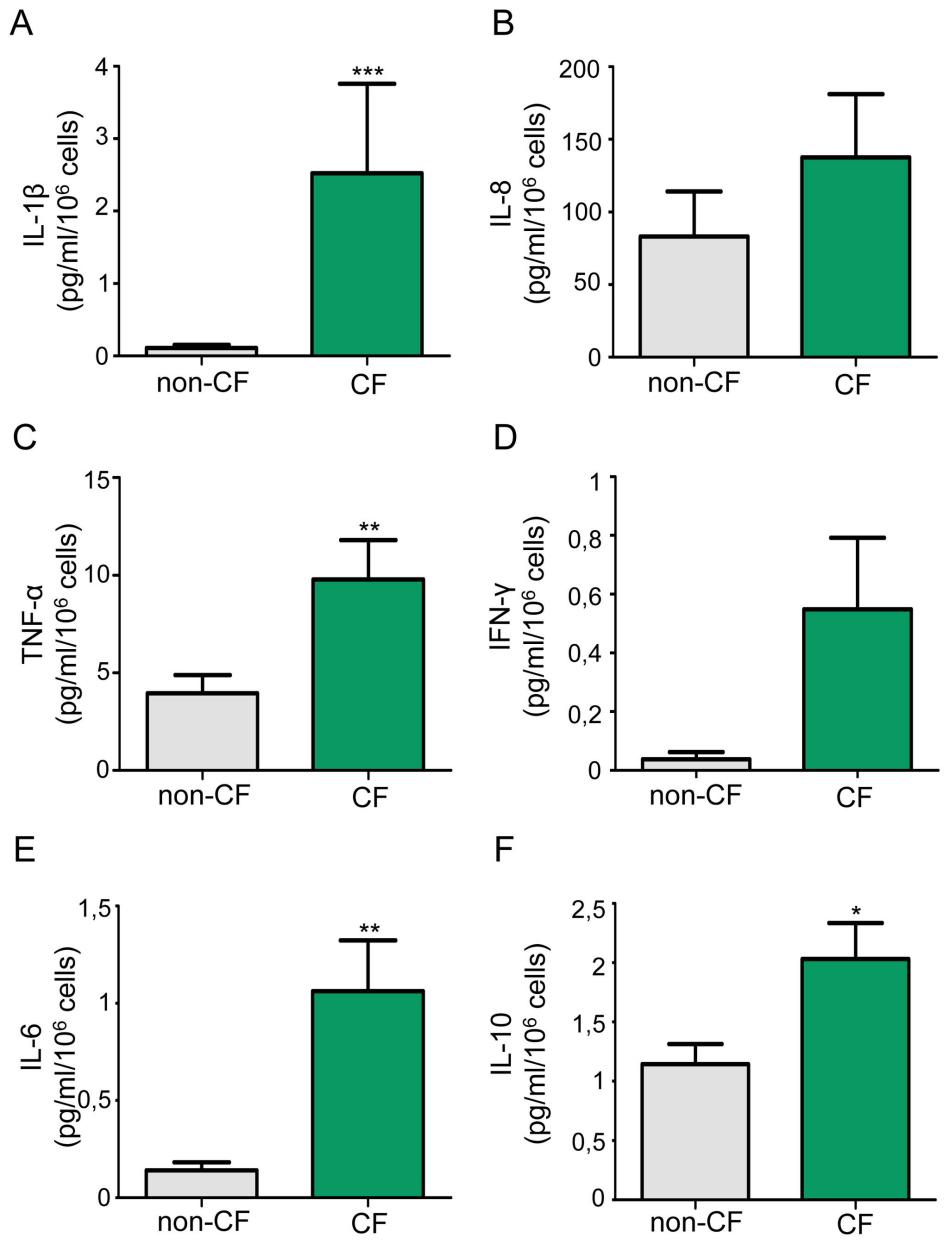
Overproduction of inflammatory cytokines in CF macrophages under basal conditions. IL 1β, IL 8, TNF-α, IFN-γ, IL-6 and IL-10 levels were measured in supernatants of non-CF and CF macrophages. (*A*) IL-1β data are shown as mean ± SEM of seven and eighteen independent experiments respectively for non-CF and CF macrophages (patients 1-3, 8, 10, 12, 14, 20-25, 32-33, 35, 37 and 42; table S1). (*B*) IL-8 data are shown as mean ± SEM of six and nineteen independent experiments respectively for non-CF and CF macrophages (patients 1-3, 8, 10-12, 14-17, 19-21, 23, 33-35 and 37; table S1). (*C to F*) TNF-α, IFN-γ, IL-6 and IL-10 data are shown as mean ± SEM of thirteen and twenty-five independent experiments respectively for non-CF and CF macrophages (patients 1-4, 6, 8-21, 32,33, 37, 42 and 43).

To determine whether macrophages participate to chronic inflammation in CF pathophysiology, we have investigated production of major pro-inflammatory cytokines/chemokines by macrophages. Under basal conditions, IL-1β was significantly overproduced in CF macrophages at mRNA level and in supernatants (Figures 1A and *S2A*). Moreover, TNF-α, IL-6 and IL-10 levels were significantly increase in CF macrophage supernatants (Figure 1*C/E/F*) whereas low levels of IFN-γ were secreted by macrophages with no significant difference between non-CF and CF macrophages (Figure 1D). At the same time, basal levels of IL-8 expression and secretion were not significantly different between CF and non-CF macrophages (Figures 1B and *S2B*). Yet, according to the FEV1 (% predicted) values the majority of patients had mild disease (FEV1≥55%, n=12). Moreover, considering the FEV1 values, CF patients with more severe disease (FEV1≤55%, n=7) showed higher but no significant increase of IL-8 secretion (162.9±58.5 pg/ml/10^6^ cells, p=0.08 *vs* non-CF) than mild disease group (122.9±61.4 pg/ml/10^6^ cells, p=0.288 *vs* non-CF). By this way, whatever the FEV1, IL-1β, IL-6, IL-10 and TNF-α secretion remains significantly overproduced. Taken together, these observations highlight an excessive inflammatory basal response of CF macrophages.

**Figure 2:**
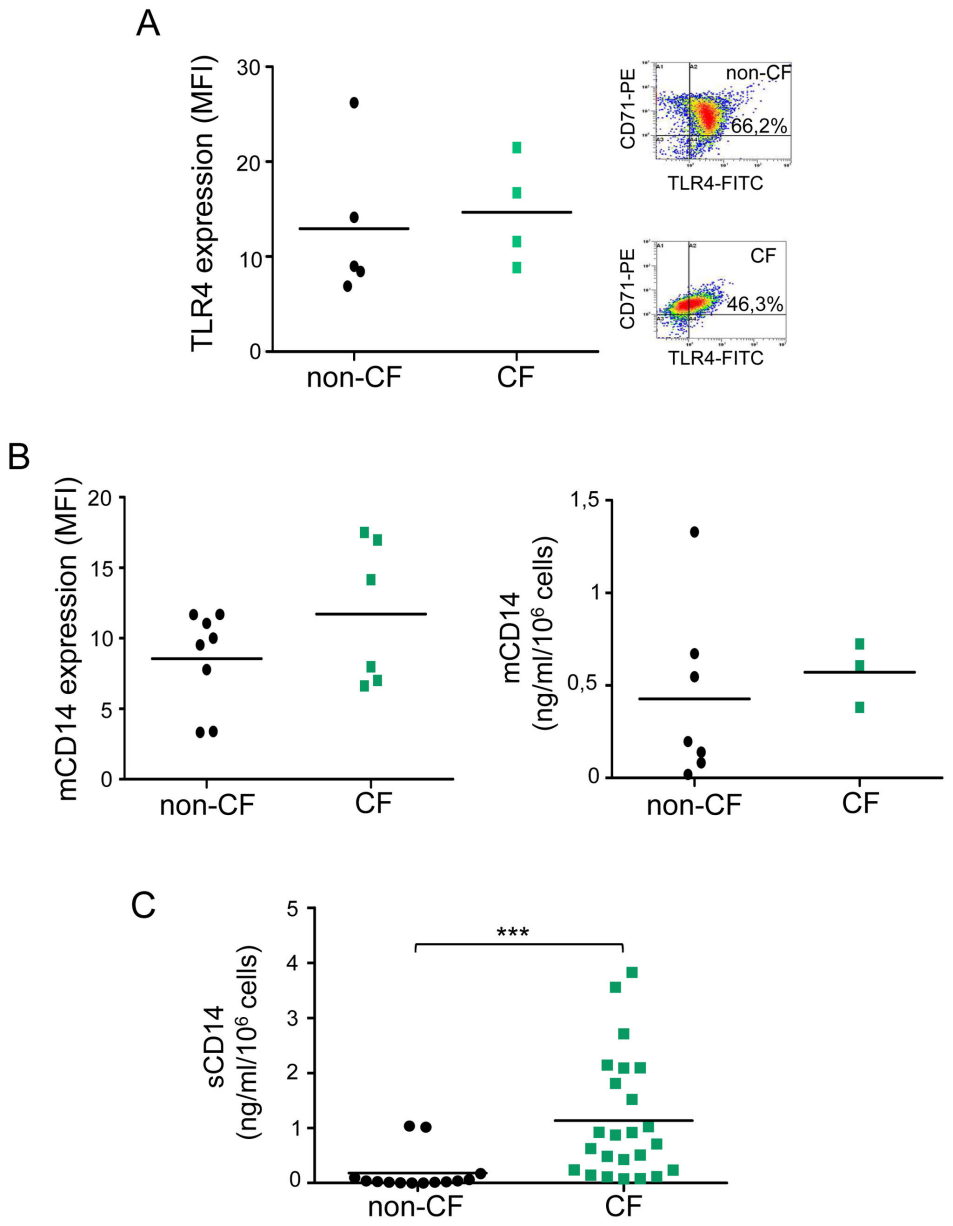
sCD14 are highly secreted by CF macrophages whereas membrane expression of TLR‑4 and mCD14 are unchanged. (*A*) *Left* *panel*: scatter plot shows TLR-4 expression on non-CF (n= 5) and CF (n=4, patients 22, 39-41; Table S1) CD71+ macrophages, analyzed by flow cytometry using an anti-TLR-4-FITC and expressed as mean fluorescence intensity (MFI, arbitrary unit of fluorescence intensity). Each symbol represents a single individual and line is the mean MFI value. Right panel: representative density plot for non-CF and CF cells. (B) Left panel: Scatter plot shows mCD14 expression on non-CF (n= 8) and CF (n=6, patients 5-8, 32 and 42; Table S1) CD71+ macrophages, analyzed by flow cytometry using an anti-CD14-FITC and expressed as MFI. Right panel: scatter plot shows mCD14 expression in non-CF (n=7) and CF (n=3, patients 7, 30 and 31; Table S1) macrophages, analyzed by cell-based ELISA. Each symbol represents a single individual and line is the mean MFI value. (C) sCD14 levels secreted by non-CF and CF macrophages were quantified by ELISA. Each symbol represents a single individual and line is the mean sCD14 concentration. Scatter plot shows fourteen and twenty-four independent experiments respectively for non-CF and CF macrophages (patients 1-4, 9, 11-25, 32, 34 and 42-43; Table S1). Mann and Whitney test: *** p<0.001 *vs* non-CF macrophages.

Presence of Toll-Like receptors (TLRs), especially TLR-4, on the cell surface of leukocytes is important in signaling pathways that activate production of pro-inflammatory cytokines. In this study, we have investigated the expression of TLR-4 and its co-activator mCD14 as well as the secretion of the soluble form (sCD14) by non-CF and CF macrophages. Concerning TLR-4 (Figure 2A) and mCD14 expression (Figure 2B), no significant difference was observed between non-CF and CF macrophages. Although a slight decrease of TLR-4 expressing cells was observed in CD71-positive CF macrophages (46.3% *vs* 66.2% in control cells) this difference was no significant. However, a sharp significant increase of sCD14 secretion by CF macrophages was observed (Figure 2C). Moreover, mCD14 cell-based ELISA (Figure 2B, right panel) allowed us to determine a sCD14/mCD14 ratio that was 44 times more important in CF (18.44) than in non-CF (0.42) macrophages. These results suggest a deregulation of TLR-4 and inflammation pathway in CF macrophages.

### CD11b and TLR-5 expressions are decreased on CF macrophage membrane

To fulfill their function effectively as possible, macrophages express on their membrane many receptors allowing pathogens recognition. As their defect could affect macrophage innate defense functions, we have studied in depth main characteristic receptors involved in bacteria detection, such as CD11b (complement C3b are recognized by CR3, an opsonic and nonopsonic phagocytic receptor), CD64 and CD16 (immunoglobulin receptors Fc-gammaRI and III respectively that recognized IgG opsonins), TLR-5 (flagellin receptor), TLR-1 and TLR-2 (pathogen-associated molecular patterns (PAMPs) receptor).

**Figure 3:**
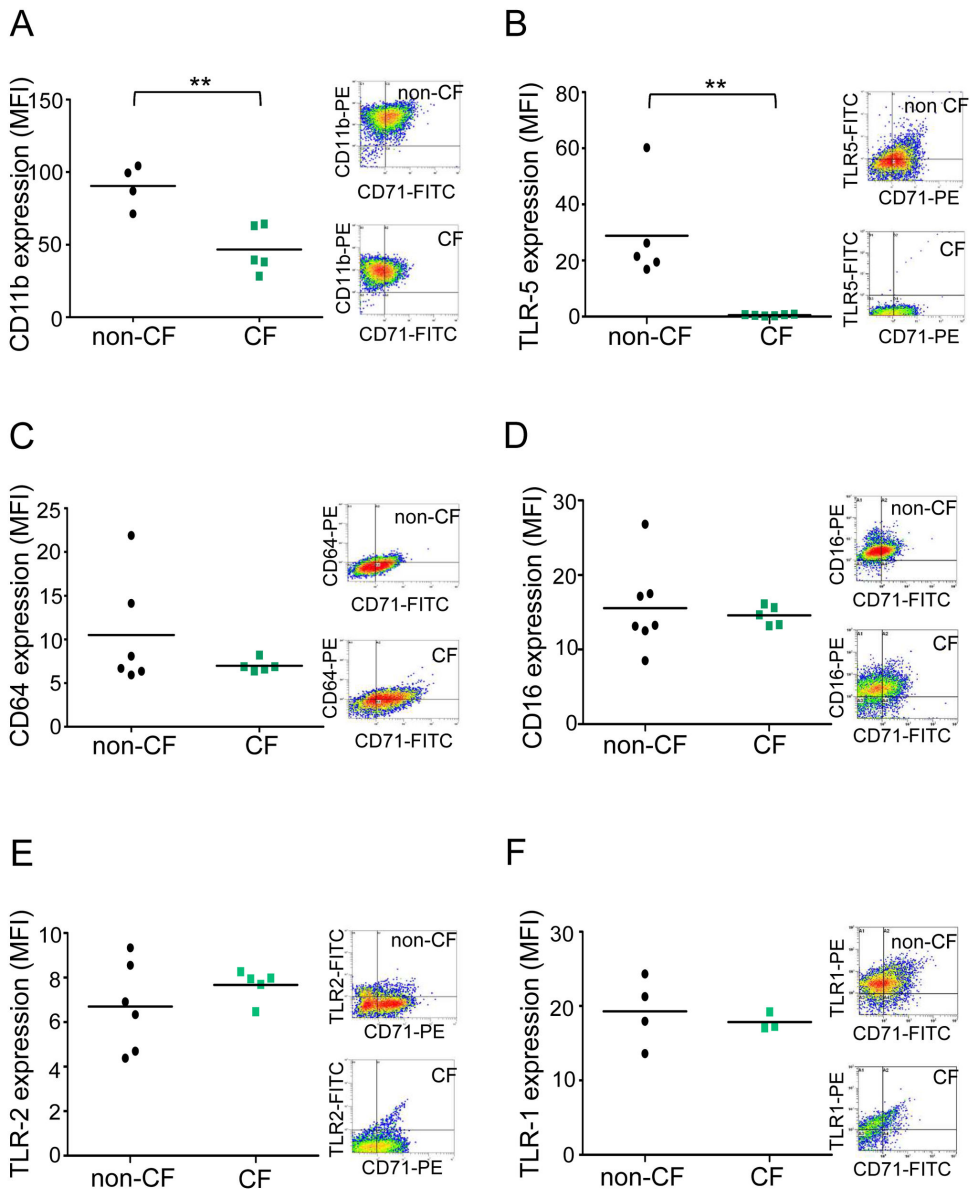
CD11b and TLR-5 expression are decreased on CF macrophage membrane. (A) Scatter plot shows CD11b expression on non-CF (n= 4) and CF (n=5, patients 1-4 and 43; Table S1) CD71+ macrophages analyzed by flow cytometry using an anti-CD11b-PE. (B) Scatter plot shows TLR-5 expression on non-CF (n= 5) and CF (n=6, patients 9, 11, 13, 18, 44 and 45; Table S1) CD71+ macrophages analyzed by flow cytometry using an anti-TLR-5-FITC. (C) Scatter plot shows CD64 expression on non-CF (n= 7) and CF (n=5, patients 3, 11, 13, 17 and 20; Table S1) CD71+ macrophages analyzed by flow cytometry using an anti-CD64-PE. (D) Scatter plot shows CD16 expression on non-CF (n= 6) and CF (n= 5, patients 9, 15, 16, 18 and 19; Table S1) CD71+ macrophages analyzed by flow cytometry using an anti-CD16-PE. (E) Scatter plot shows TLR-2 expression on non-CF (n= 6) and CF (n= 5, patients 6, 9, 22, 29 and 35; Table S1) CD71+ macrophages analyzed by flow cytometry using an anti-TLR-2-FITC. (F) Scatter plot shows TLR-1 expression on non-CF (n= 4) and CF (n= 3, patients 33, 42 and 46; Table S1) CD71+ macrophages analyzed by flow cytometry using an anti-TLR-1-PE. Results are expressed as mean fluorescence intensity (MFI, arbitrary unit of fluorescence intensity). Each symbol represents a single individual, and line is the mean MFI value. Right panels show representative density plot for non-CF and CF cells. Mann and Whitney test: ** p<0.01 *vs* non-CF macrophages.

Flow cytometry analysis in CD71-positive cells showed a strong significant decrease of CD11b expression (Figure 3A) but no significant decrease of CD64, CD16, TLR-2 and TLR-1 expressions (Figure 3*C/D/E/F*) suggesting a loss of pathogen recognition by CF macrophages involving more specifically CD11b. Moreover, membrane TLR-5 (Figure 3B) was not expressed in CF macrophages in comparison to non-CF macrophages highlighting a less effective recognition of mobile pathogens such as *P. aeruginosa* during CF.

In order to determine the specificity of variations observed in CF patients, we analyzed the expression of TLR-5/-4/-1/-2 in non-CF CD71^+^ macrophages and the IL-1β and TNF-α secretion in the presence of their respective agonists (flagellin, LPS, Pam3CSK4 and LipoTeichoic Acid (LTA)) (Figure S3). By this way, we did not observed any significant decrease of TLRs expression after agonist exposure (Figure S3, *left panel*) suggesting that TLR-5 decrease observed in CF patients was specific of this disease. Moreover, in all cases, agonist exposure led to increase of IL-1β and TNF-α secretion by macrophages showing that our macrophages express functional TLRs (Figure S3, *right panel*).

### Decreased phagocytosis capacity in CF macrophages

After demonstrating that receptors involved in pathogens recognition are altered in CF macrophages, we wondered if the phagocytic capacity of macrophages is also distort in CF. We thus evaluated this function by two methods using two gram-negative pathogens bacteria.

**Figure 4:**
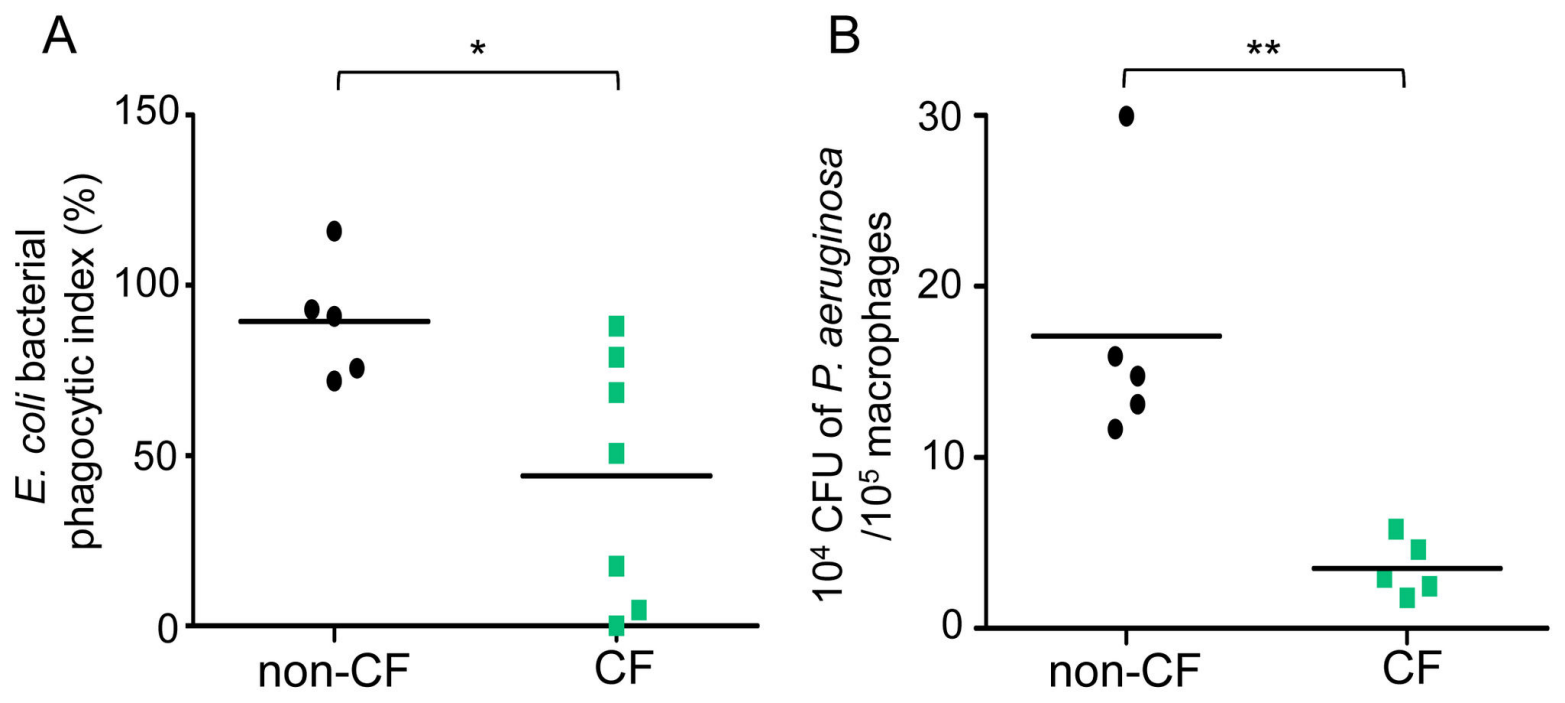
Phagocytosis is sharply decreased in CF macrophages. (*A*) Non-CF and CF macrophages were incubated with heat-inactivated *E. coli*-fluorescein for 2 hours (MOI: 100). Scatter plot shows *E. coli* bacterial phagocytic index (%) (experimental reading minus negative-control reading / positive-control reading minus negative-control reading) of non-CF (n=5) and CF macrophages (n=7, patients 11, 22, 26-29 and 38; Table S1). Each symbol represents a single individual, and line is the mean bacterial phagocytic index. (B) Lived-P. aeruginosa were added to non-CF and CF macrophage cultures during 1 hour (MOI: 100). The intracellular bacteria were evaluated by lysis cells and count on agar plates after a one hour gentamycin (150 µg/ml) exposure. Each symbol represents a single individual, and line is the mean CFU. Scatter plot shows five independent experiments for non-CF and CF macrophages (patients 13, 26 and 29-31; Table S1). Mann and Whitney test: * p< 0.05, ** p<0.01 *vs* non-CF macrophages.

In the one hand, we determined phagocytosis capacity of primary human macrophages from healthy subjects and CF patients towards heat-inactivated *E. coli* linked to fluorescein. In that context, CF macrophages present a significant defect of phagocytosis function *vs* non-CF macrophages (67.13% of decrease) (Figure 4A). Treatment with cytochalasin D was used as a positive control for each experiment (Figure S4).

In the second hand, we studied the phagocytosis of the opportunistic gram-negative flagelled bacteria *P. aeruginosa* chronically infecting lung from CF patients [14] and more especially adults [15]. A significantly sharp decrease of CFU bacteria number/10^5^ macrophages in CF macrophages was observed (79.37% of decrease) (Figure 4B), highlighting once again the decrease of phagocytosis in CF macrophages.

### Influence of CFTR inhibition on non-CF macrophage functions

**Figure 5:**
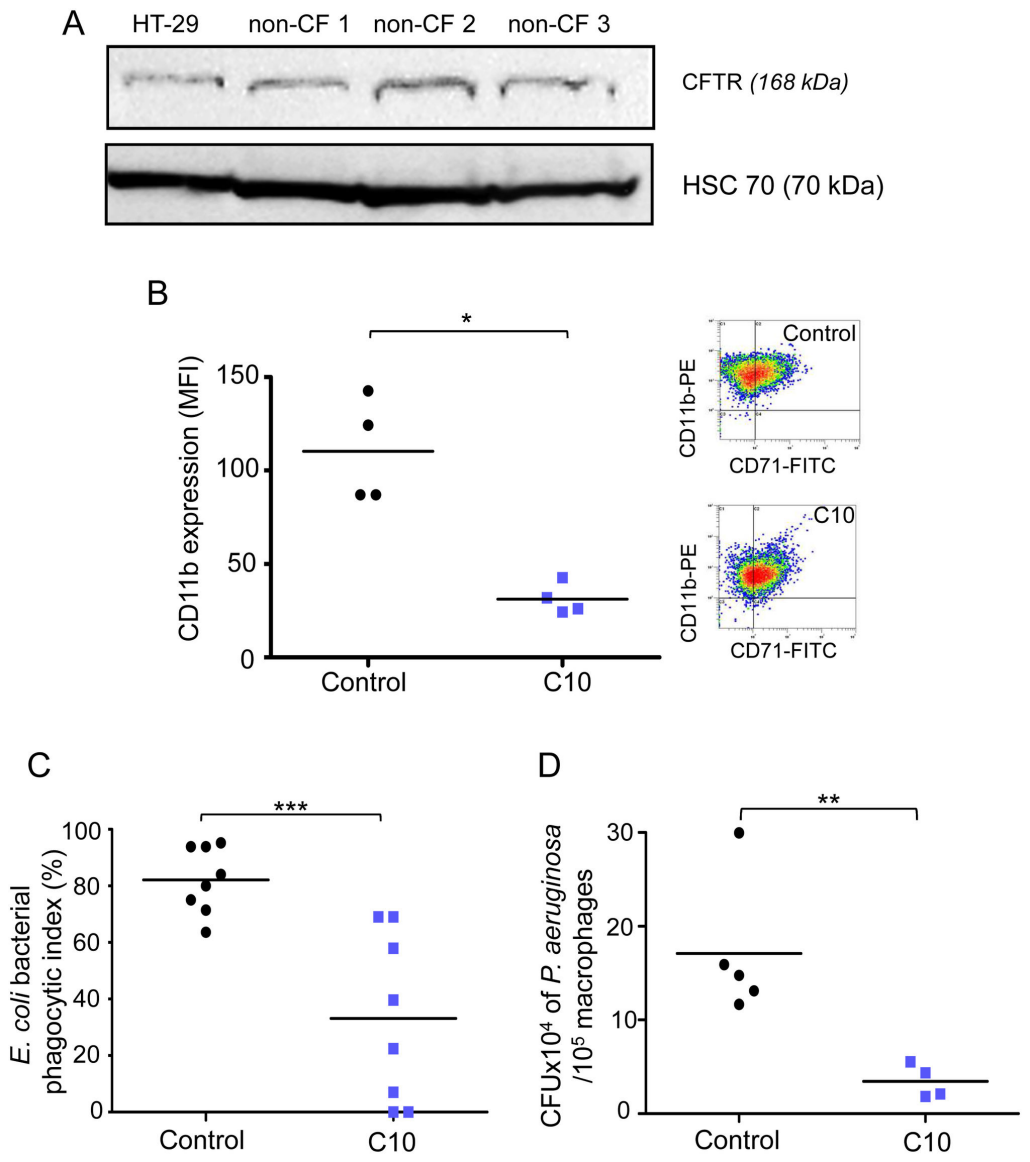
CD11b expression and phagocytosis are decreased in non-CF macrophages treated by CFTR_inh172_. (*A*) CFTR expression was checked by western-blot on total membrane fraction from non-CF macrophages (three separate representative experiments: non-CF1, non-CF2 and non-CF3) and HT29 (Human colon adenocarcinoma grade II cell line) as reference. Equal loading was controlled for by HSC-70 detection. (*B*-*D*) Non-CF macrophages were treated with CFTR_inh-172_ (C10, 10 µM) for 72h. (*B*) Scatter plot shows CD11b expression on non-CF CD71^+^ macrophages with (C10, n=4) or without (control, n=4) CFTR_inh172_, analyzed by flow cytometry using an anti-CD11b-PE and expressed as mean fluorescence intensity (MFI, arbitrary unit of fluorescence intensity). Each symbol represents a single individual, and line is the mean MFI value. Right panel shows representative density plot for non-CF cells. (*C*) Non-CF macrophages were incubated with heat-inactivated *E. coli*-fluorescein for 2 hours (MOI: 100). Scatter plot shows *E. coli* bacterial phagocytic index (%) (experimental reading minus negative-control reading / positive-control reading minus negative-control reading) of non-CF macrophages with (C10, n=8) or without (control, n=8) CFTR_inh-172_. Each symbol represents a single individual, and line is the mean bacterial phagocytic index. (*D*) Lived-*P*. *aeruginosa* were added to non-CF and CF macrophage cultures during 1 hour (MOI: 100). The intracellular bacteria were evaluated by lysis cells and count on agar plates after a one hour gentamycin (150 µg/ml) exposure. Each symbol represents a single individual and line is the mean CFU. Scatter plot shows five independent experiments for non-CF and CF macrophages with (C10, n=4) or without (control, n=5) CFTR_inh172_. Each symbol represents a single individual and line is the mean CFU. Mann and Whitney test: * p<0.05; ** p<0.01 and *** p<0.001 *vs* non-CF macrophages.

In order to elucidate the relation between our previous results showing macrophage dysfunctions and CFTR defect, we have pharmacologically inhibited CFTR function in non-CF macrophages. For this purpose, mature form of CFTR protein was characterized by Western-blotting in non-CF macrophages (Figure 5A), demonstrating that our model of human monocyte-derived macrophages express CFTR protein.

**Figure 6:**
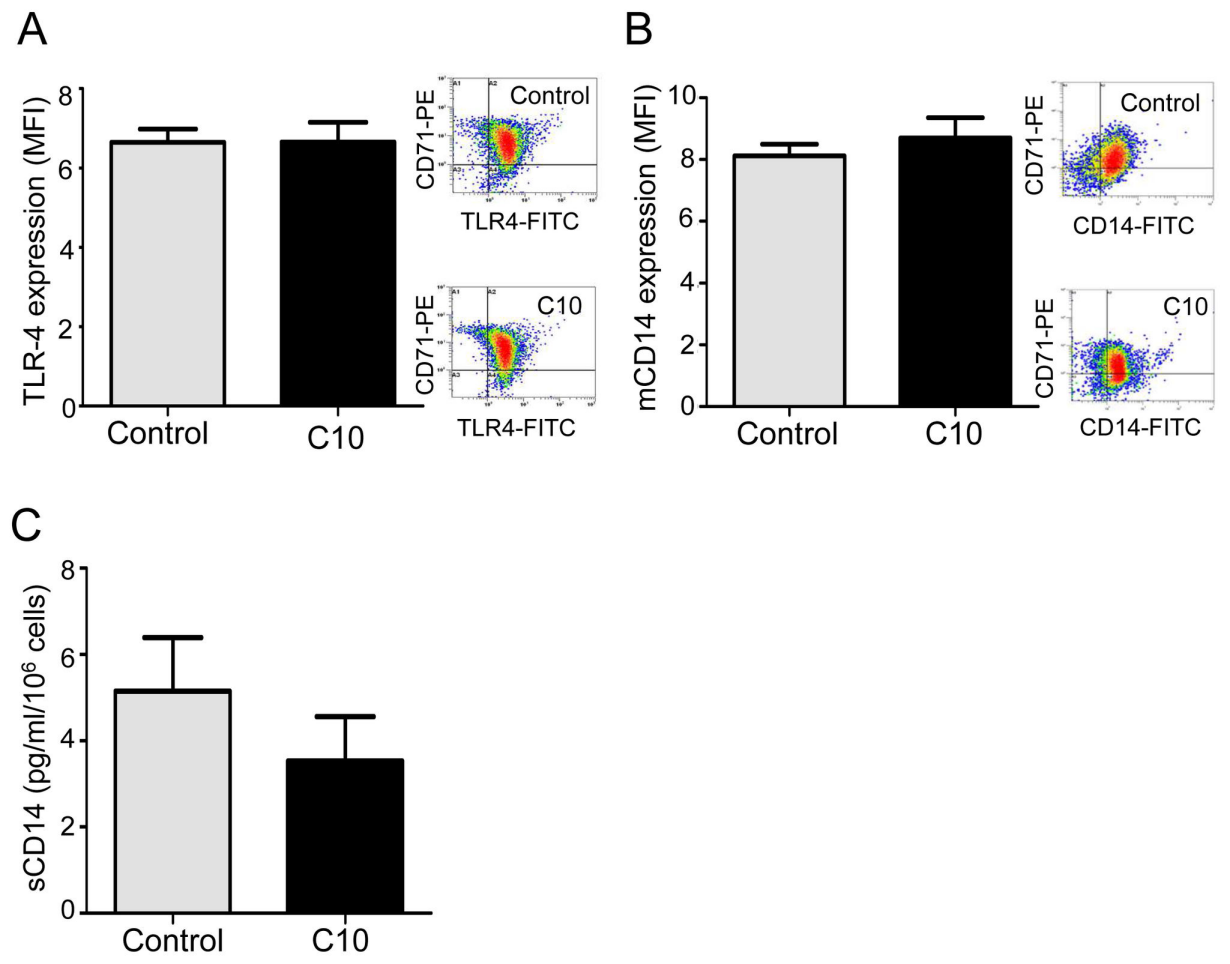
CFTR_inh-172_ does not influence TLR-4, mCD14 expression and sCD14 levels in non-CF macrophages. Non-CF macrophages were treated with CFTR_inh-172_ (C10, 10 µM) for 72h. (*A*) TLR4 expression on non-CF CD71^+^ macrophages with (C10, n=5) or without (control, n=4) CFTR_inh172_ analyzed by flow cytometry using an anti-TLR-4-FITC and expressed as mean fluorescence intensity (MFI, arbitrary unit of fluorescence intensity). Right panel shows representative density plot for each group (control and C10). (*B*) mCD14 expression by non-CF CD71^+^ macrophages with (C10, n=4) or without (control, n=7) CFTR_inh172_ analyzed by flow cytometry using an anti-CD14-FITC and expressed as MFI. Right panel shows representative density plot for each group (control and C10). (*C*) sCD14 level secreted by non-CF macrophages with (C10, n=8) or without (control, n=8) CFTR_inh172_ was quantified by ELISA.

**Figure 7:**
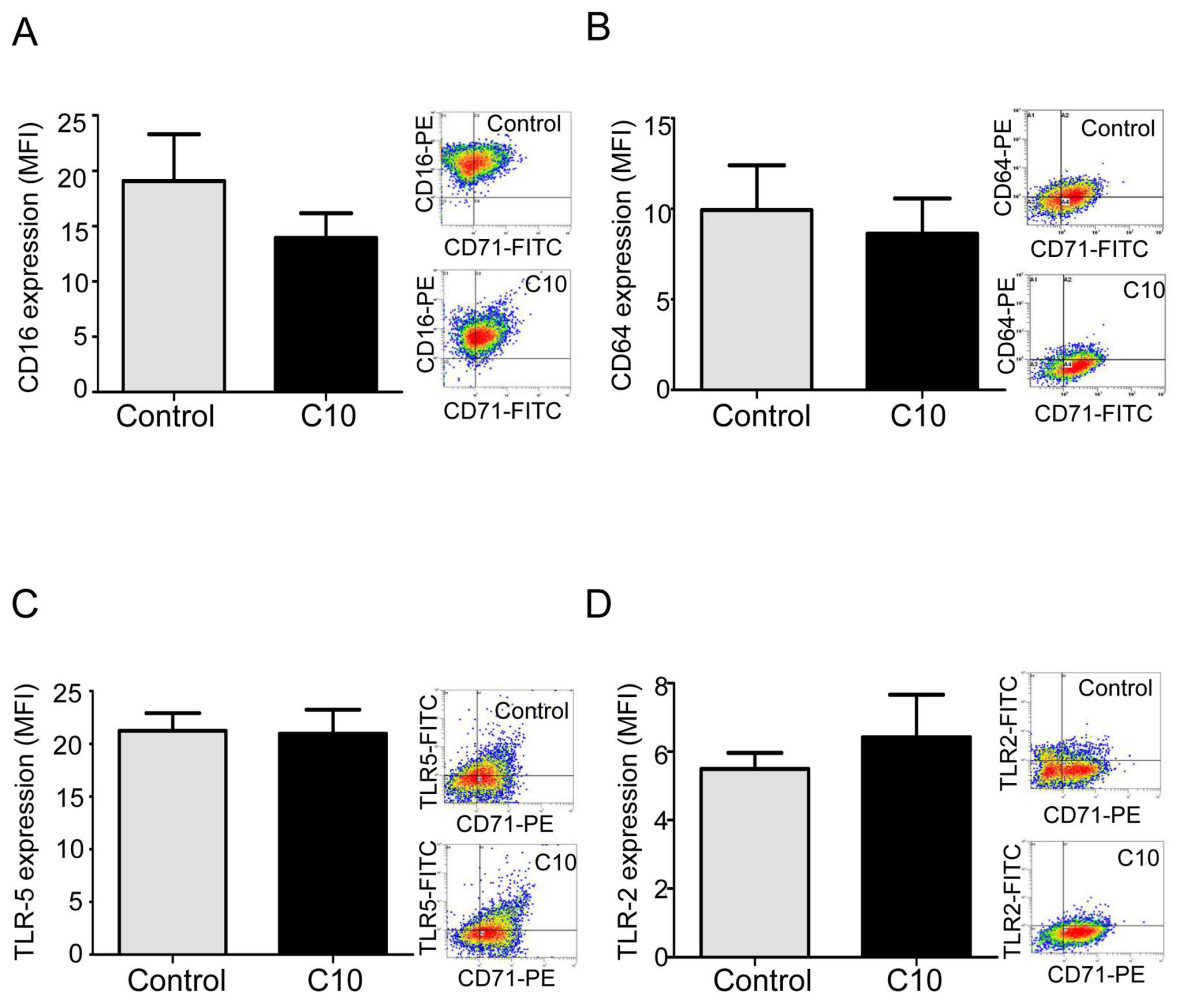
CFTR_inh-172_ does not influence CD16, CD64, TLR-5 and TLR-2 expression by non-CF macrophages. (*A*) CD16 expression on non-CF CD71^+^ macrophages with (C10, n=3) or without (control, n=3) CFTR_inh172_ analyzed by flow cytometry using an anti-CD16-PE and expressed as mean fluorescence intensity (MFI, arbitrary unit of fluorescence intensity). (*B*) CD64 expression on non-CF CD71^+^ macrophages with (C10, n=4) or without (control, n=4) CFTR_inh172_ analyzed by flow cytometry using an anti-CD64-PE and expressed as MFI. (*C*) TLR-5 expression on non-CF CD71^+^ macrophages with (C10, n=4) or without (control, n=4) CFTR_inh172_ analyzed by flow cytometry using an anti-TLR-5-FITC and expressed as MFI. (*D*) TLR-2 expression on non-CF CD71^+^ macrophages with (C10, n=4) or without (control, n=4) CFTR_inh172_ analyzed by flow cytometry using an anti-TLR-2-FITC and expressed as MFI. Right panels show representative density plot for each group (control and C10).

Therefore, we determined if CFTR defect during CF has an impact on macrophage functions. In this way, in non-CF macrophages, we inhibited CFTR function with CFTR_inh-172_ at the appropriate concentration previously shown as specific against CFTR chlore efflux (10 µM) [5,16,17], and compared if modifications of macrophage characteristics occur. Interestingly CFTR inhibition, that did not affect viability of macrophages (Figure S5A), led to a significant decrease of CD11b expression (Figure 5B) as well as related phagocytosis (Figure 5C/D) but did not influence TLR-4, mCD14, CD16, CD64, TLR-5 and TLR-2 expressions as well as sCD14 secretion (Figures 6 and 7). sCD14/mCD14 ratio was also unchanged after CFTR inhibition (ratio was 0.012 and 0.011 for control and CFTR_inh172_ 10 µM respectively). Elsewhere IL-1β production was not influenced by CFTR inhibition (Figure S5B).

The alterations of macrophage functions observed in our study seem thus not only to be associated with CFTR inhibition, but may also be linked to chronic hyper-inflammatory status of CF immune cells.

## Discussion

Chronic lung inflammation, which is clearly associated with infection, represents the main cause of morbidity/mortality in CF patients [18]. Today, inability to efficiently resolve bacterial inflammation/infections in CF has been imputed to defect in defense mechanisms mediated by epithelial cells and neutrophils [19–21]. Macrophages may however play a significant role in the initiating stages of this disease, *via* an inability to act as a pathogen suppressor cell. Our study was performed with primary human macrophages obtained by GM-CSF-mediated differentiation of blood monocytes allowing a similar alveolar macrophages phenotype [13,22]. In this work, we considered CF *vs* non-CF macrophages in order to determine differences in their functions related to chronic inflammation/infection and the impact of CFTR pharmacological inhibition. Using nonopsonic conditions, thus mimicking resting and naïve conditions in the alveolar space, we showed an alteration of CF macrophage functions.

Macrophages coordinate inflammatory responses by secreting inflammatory mediators including TNF-α, IL-1β, IL-6, IFN-γ, IL-10 and IL-8. These inflammatory mediators have diverse physiological effects potentially important to the pathogenesis of lung exacerbations in CF, including the generation of fever, the recruitment of inflammatory effector cells and the shaping of T cell responses resulting in chronic infection/inflammation [23–26]. Increased level of these cytokines was shown in plasma, sputum and bronchoalveolar lavage from CF patients [25,27–29] Under basal conditions IL-8 levels were increased in human CF plasma and sputum and also related to the severity of the pathology [24,30]. However it was also shown similar IL-8 production in CF patients with mild disease (FEV1 ≥ 55%) compared to healthy control whereas CF patients with more severe disease (FEV1 ≤ 55%) have a higher basal IL-8 secretion [24]. In agreement with these previous studies we have shown that human CF macrophages overproduce IL-1β whereas IL-8 basal level and mRNA expression was not significantly increased, yet observing higher IL-8 levels in CF patients with FEV1 ≤ 55%.

Regarding CD14 expression, a strong increased secretion of the soluble form sCD14 was observed in our study whereas no change of mCD14 expression was shown between CF and non-CF macrophages. These data are in agreement with previous studies showing that elevated concentration of sCD14 was found in serum of patients with bacterial infections [39,40] as well as in other chronic inflammatory diseases such as lupus erythematosus [41] or pneumonia [42]. Although the mechanism of sCD14 release from cells is not completely understood, the involvement of mCD14 endocytosis [43] and of proteolytic cleavage by elastase present at large amounts in CF airways [44] have been suggested. Moreover, previous study showed that sCD14 could be considered as an acute-phase protein [45]. In multiple sclerosis, sCD14 was suggested to be an antagonist of TLR-4 signaling at low concentrations but could promote pro-inflammatory cytokines release at high concentrations [46]. These entire hypotheses remain to be demonstrated and are under investigation.

Because TLR-4 and TLR-1/TLR-2 are critical in mediating Gram-positive and Gram-negative-induced cell signaling respectively, we investigated their expression in non-CF and CF macrophages. Our phenotypic study of human primary macrophages reveals that expressions of early PAMPs receptors, TLR-4 and TLR-1/TLR-2, are not significantly changed between CF and non-CF macrophages, suggesting that their expressions in macrophages are not involved in impaired defense against pathogens in CF. This observation gives new insights compared to previous studies showing that TLR-4 expression was significantly reduced in CF bronchial epithelial cell line CFBE41o, contributing to chronic bacterial infection of CF airways [31]. However it was also demonstrated that reduction of TLR-4 and TLR-2 expressions was only observed in CF bronchial epithelium but not in CF human immune cells [32], in agreement with our results in CF macrophages. Furthermore, in healthy subjects TLR-4 is mainly expressed by monocytic cells, whereas in patients with CF the infiltrating neutrophils are the major source of TLR-4 (38). Regarding TLR-5, its involvement has been shown in the recognition of flagelled bacteria like *P. aeruginosa* and it is thus considered as a critical factor for alveolar macrophage phagocytosis [33]. In our study, CF macrophages did not express TLR‑5 on their membrane compared to non-CF macrophages, suggesting that this toll-like receptor is one of the limiting factors for *P. aeruginosa* recognition and phagocytosis in CF.

Furthermore, we observed an important decrease of CD11b expression as well as phagocytosis in CF macrophages. CD11b or CR3, an heterodimer integrin composed of α- and β-subunits, is the major receptor for opsonic phagocytosis of many bacteria [34,35]. Previous studies have also shown nonopsonic ingestion of microbial pathogens (e.g *Mycobacterium tuberculosis* [36,37], and *Leishmania* [38]) through CR3-mediated phagocytosis. According to these data and the fact that CD11b and phagocytosis are diminished in CF macrophages, we strongly suggested here that *P. aeruginosa* phagocytosis in CF occurs through a nonopsonic manner involving especially CD11b. Beside, expression of Fc receptors CD16 and CD64, which recognize the immunoglobulin Fc domain constituting an opsonin, are unchanged in our model of CF macrophages. These results underscore the key role of nonopsonic ingestion of bacteria in CF macrophages and validate the pivotal role of CD11b. Together with the fact that TLR-5 is not expressed on CF macrophages, all these results allow to understand how infection sets in CF patients, notably for *P. aeruginosa* pathogen.

Our results showed that phagocytosis capacity is altered in CF macrophages. In contrast, previous published data exposed that murine and human CF macrophages have defective bacterial killing without impaired internalization [9,17]. As far as murine alveolar macrophages were concerned [17], the use of murine model does not accurately reflect the pathophysiology of human CF disease because no respiratory impairment was observed in mice. Differences observed with study using human monocytes-derived macrophages [9] may be attributed to experimental procedure for monocytes selection and differentiation leading to different macrophage phenotype. In fact, the selection of monocytes was realized with CD14-positive selection and differentiation with M-CSF [9]. These macrophages have a morphology of extended cells and cell surface antigen are not similarly expressed, that lead to differences in their function whereas in our model, selection of monocytes by adhesion followed by differentiation with GM-CSF lead to alveolar phenotype macrophages mostly involved in lung defense against pathogens [47].

We can therefore consider that CF macrophages are unable to properly recognize pathogens, and that their decreased phagocytic capacity leads to persistent infection. Chronic infection induces a continuous stimulation of macrophages, contributing to unremittingly pro-inflammatory cytokine overproduction. This continuous cytokine secretion then strongly disturbs macrophage functions. A vicious cycle between inflammation and infection appears. Our data sharply suggest that changes of macrophage functions involved in the inflammation resolution could be responsible for chronic infection. However, which of inflammation or infection appears first in CF is still a pending question. In this context, it remains to explore if the inhibition of CFTR function modifies macrophage physiology that can led to alteration of its function and thus to chronic inflammation and infection. As demonstrated in previous studies we confirmed the presence of CFTR protein in our model of human primary macrophages. Furthermore these studies clearly showed that this protein was functional in macrophages [9,17]. We confirmed this point in our study and showed that pharmacological inhibition of CFTR in non-CF macrophages strongly decreased CD11b membrane expression and phagocytosis function. These results are in accordance with previous works showing that in CF neutrophils, CFTR defect promotes the decrease of CD11b receptor [48]. It was also shown that epithelial cells use CFTR as a receptor for internalization of *P. aeruginosa* through endocytosis and subsequent removal of bacteria from the airway [4]. Overall, our data indicate that CFTR in macrophage could be associated to phagocytosis dysfunction.

In summary, we describe a pro-inflammatory phenotype of human CF macrophages secreting higher level of sCD14 and cytokines, with decreased membrane expression of CD11b and TLR-5 leading to impaired phagocytosis and altered clearance of pathogens. The latter induces non-resolution of inflammation/infection by CF macrophages, thereby inducing an exaggerated pro-inflammatory response. Our results provide new insights into what might be markers of poor prognosis in CF patients, i.e reduction of CD11b and TLR-5 expression, as well as the sharp increase of sCD14 production. Detection of such biomarkers may be relevant in order to drive diagnostic, preventive and curative strategies during CF. In addition, our study contributes to extend understanding concerning the crucial role of macrophage in the CF lung pathophysiology, and its implication in the low defense capacity of CF patients against pathogens.

## Supporting Information

Figure S1 **Characterization of human primary macrophages.** Flow cytometric graph for CD71 membrane expression using an anti-CD71-FITC (isotypic control, grey pick) on non-CF monocytes (white pick, solid line) and non-CF (white pick, black dotted line) or CF macrophages (white pick, green dotted line). Results are representative of five, twenty-four and seventeen independent experiments respectively for monocytes, non-CF and CF macrophages (patients 1-9, 11, 13 and 15-20; table S1).
(TIF)

Figure S2 **mRNA expression of inflammatory cytokines in CF *vs* non-CF macrophages under basal conditions.** mRNA levels were determined by RT-qPCR. Data are expressed relatively to mRNA level found in non-CF cells and are shown as mean ± SEM of four independent experiments (patients 23 and 34-36; table S1). Mann and Whitney test: * p< 0.05 *vs* non-CF macrophages.
(TIF)

Figure S3 **Effect of TLRs agonists on TLRs expression (left panel) and IL-1β and TNF-α secretion (right panel) in non-CF CD71^+^ macrophages.** Expression was analyzed by flow cytometry and *expressed* *as* mean fluorescence intensity (MFI, arbitrary unit of fluorescence intensity). IL-1β and TNF-α level were assessed by ELISA. Data are shown as mean ± SEM of four independent experiments. Mann and Whitney test: * p<0.01 *vs* control for IL-1β and § p<0.01 *vs* control for TNF-α.
(TIF)

Figure S4 **Inhibition of phagocytosis by cytochalasin D in non-CF macrophages.** Cells were incubated for 16 h with cytochalasin D (10 µM) before 2 hours incubation with heat-inactivated *E. coli*-fluorescein (MOI: 100). Results are expressed as percentages as follows: (experimental reading minus negative-control reading / positive-control reading minus negative-control reading) x 100, and are scatter plot with mean of six independent experiments respectively for control and cytochalasin D-treated non-CF macrophages. Mann and Whitney test: ** p<0.01 *vs* control.
(TIF)

Figure S5 Viability of non-CF macrophages treated by CFTR_inh-172_ (*A*). Non-CF macrophages were treated with CFTR_inh-172_ at 1 to 50 µM (72 h). 100 µl of MTT solution (5 mg/ml in PBS, 3-(4,5-dimethylthiazol-2-yl)-2,5-diphenyltetrazolium bromide, Sigma-Aldrich, Saint-Quentin-Fallavier, France) were added into each well and cells were incubated at 37°C and 5% CO_2_ for 2 hours. The medium was removed and 100 µl of DMSO was added into each well. The plate was gently rotated on an orbital shaker for 10 min to completely dissolve the precipitation. The absorbance was detected at 540 nm with a microplate reader associated with Genesis software (LabSystems Spectrophotometer, Cambridge, UK). (*B*) **IL-1β levels were measured in supernatants of non-CF macrophages treated or not with CFTR_inh-172_**. IL-1β data are shown as mean ± SEM of eight independent experiments.
(TIF)

File S1 Supplemental Information.
(DOCX)

Table S1 Characteristics of CF patients.
(DOC)
