# Supplementary figures and images for: Impaired Functions of Macrophage from Cystic Fibrosis Patients: CD11b, TLR-5 Decrease and sCD14, Inflammatory Cytokines Increase
Source: PLoS One. 2013 Sep 30;8(9):e75667. doi: 10.1371/journal.pone.0075667 (PMC3787056; doi:10.1371/journal.pone.0075667)

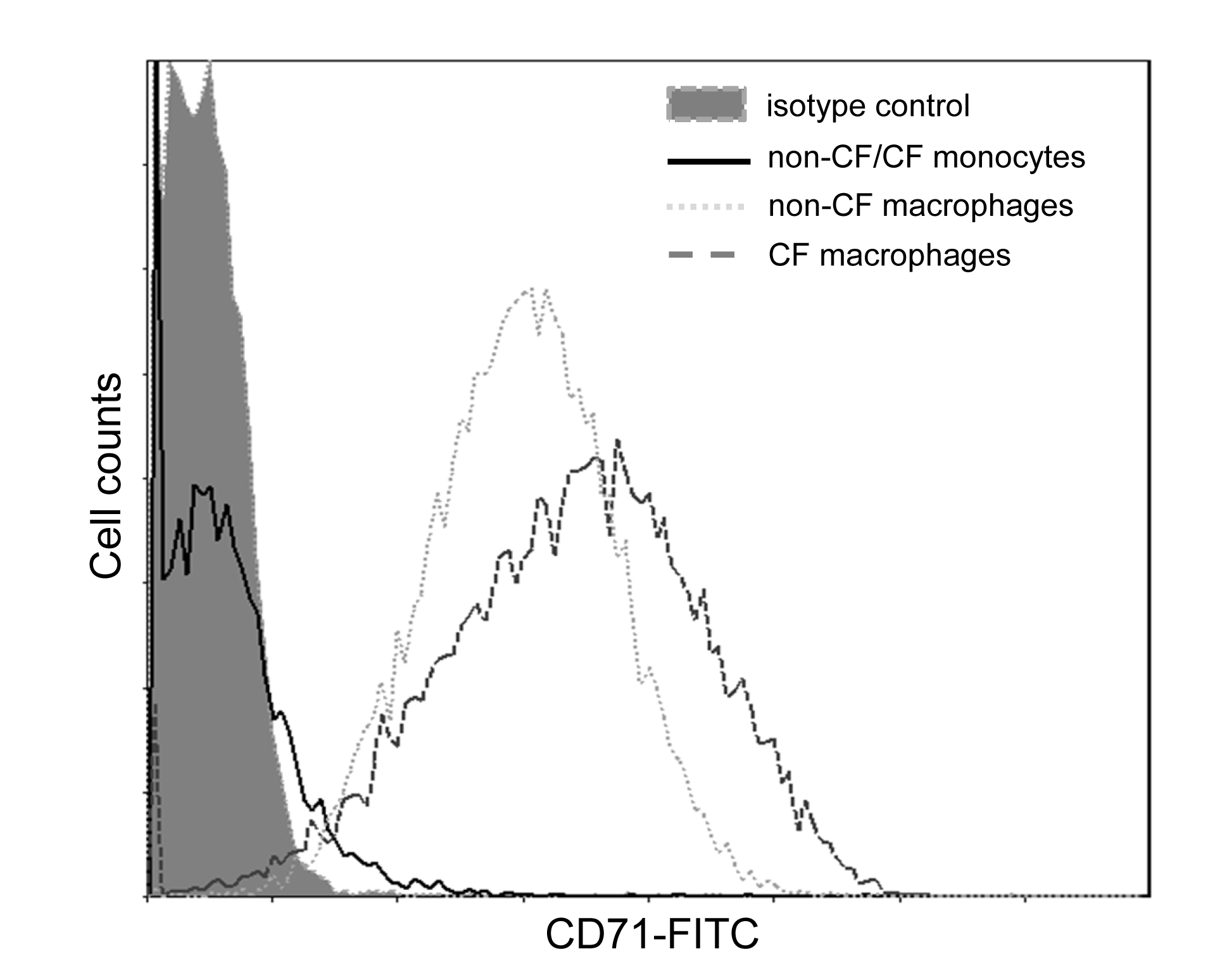

Supplement: Figure S1 — Characterization of human primary macrophages. Flow cytometric graph for CD71 membrane expression using an anti-CD71-FITC (isotypic control, grey pick) on non-CF monocytes (white pick, solid line) and non-CF (white pick, black dotted line) or CF macrophages (white pick, green dotted line). Results are representative of five, twenty-four and seventeen independent experiments respectively for monocytes, non-CF and CF macrophages (patients 1-9, 11, 13 and 15-20; table S1). (TIF) [file pone.0075667.s001.tif]

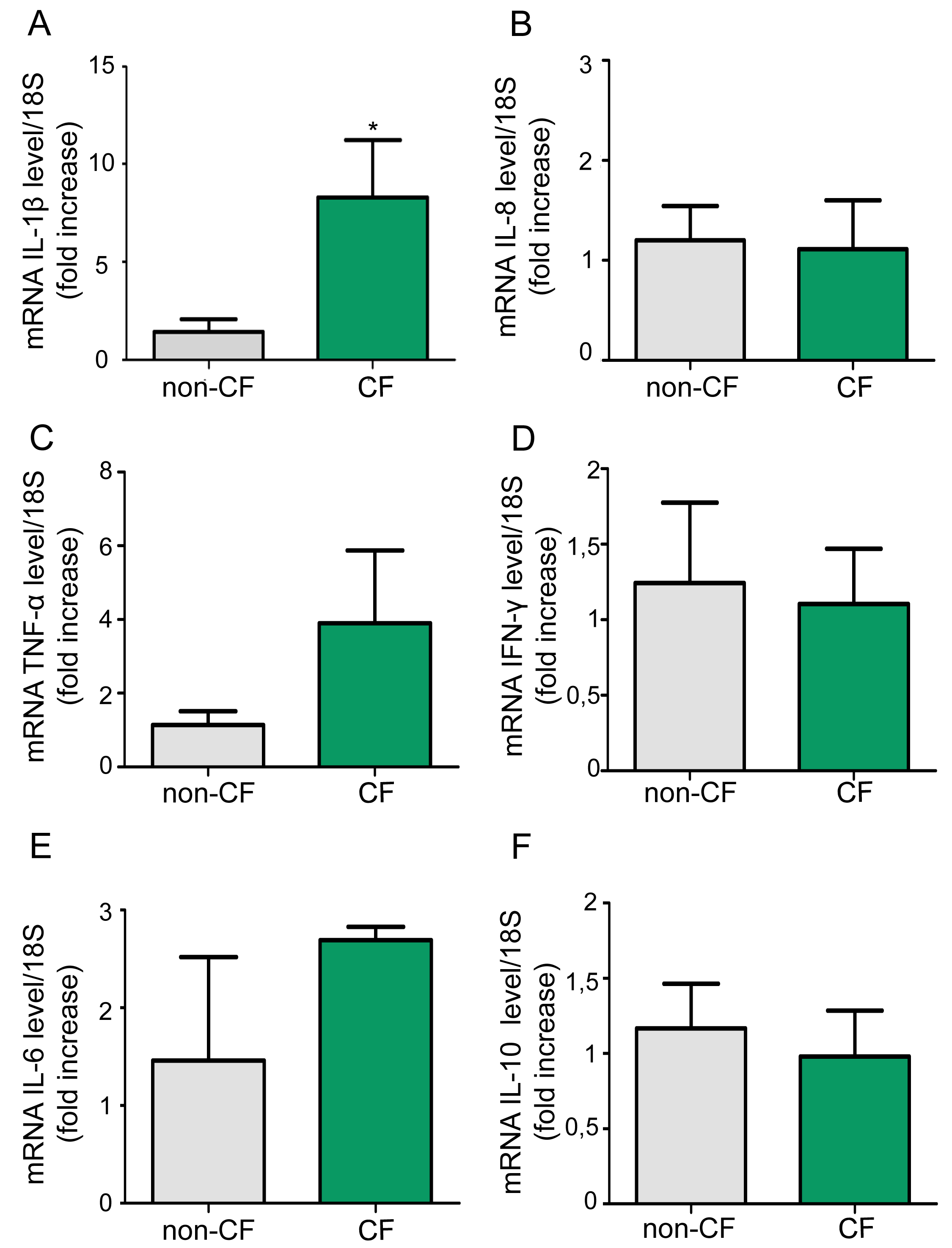

Supplement: Figure S2 — mRNA expression of inflammatory cytokines in CF vs non-CF macrophages under basal conditions. mRNA levels were determined by RT-qPCR. Data are expressed relatively to mRNA level found in non-CF cells and are shown as mean ± SEM of four independent experiments (patients 23 and 34-36; table S1). Mann and Whitney test: * p< 0.05 vs non-CF macrophages. (TIF) [file pone.0075667.s002.tif]

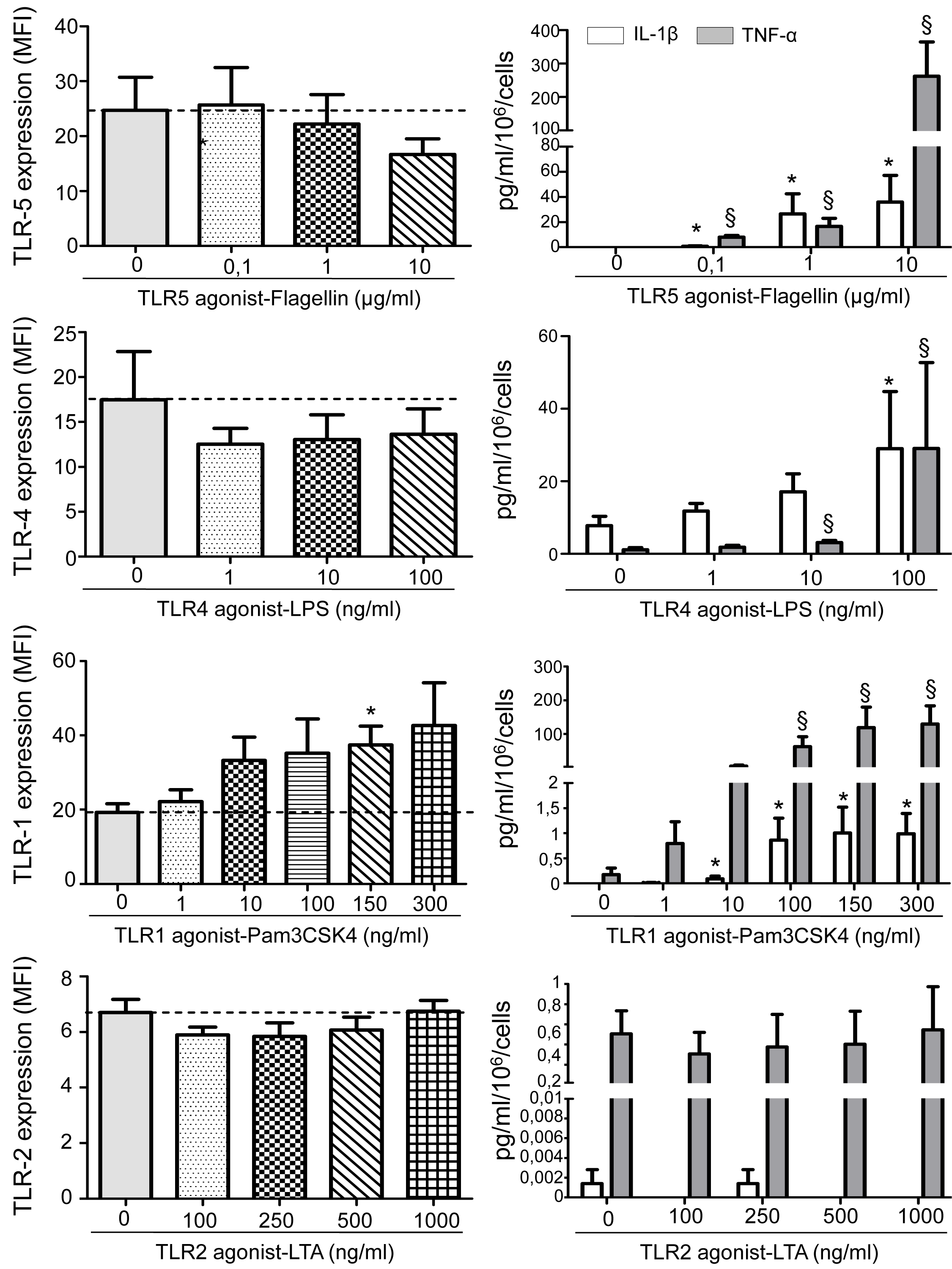

Supplement: Figure S3 — Effect of TLRs agonists on TLRs expression (left panel) and IL-1β and TNF-α secretion (right panel) in non-CF CD71+ macrophages. Expression was analyzed by flow cytometry and expressed as mean fluorescence intensity (MFI, arbitrary unit of fluorescence intensity). IL-1β and TNF-α level were assessed by ELISA. Data are shown as mean ± SEM of four independent experiments. Mann and Whitney test: * p<0.01 vs control for IL-1β and § p<0.01 vs control for TNF-α. (TIF) [file pone.0075667.s003.tif]

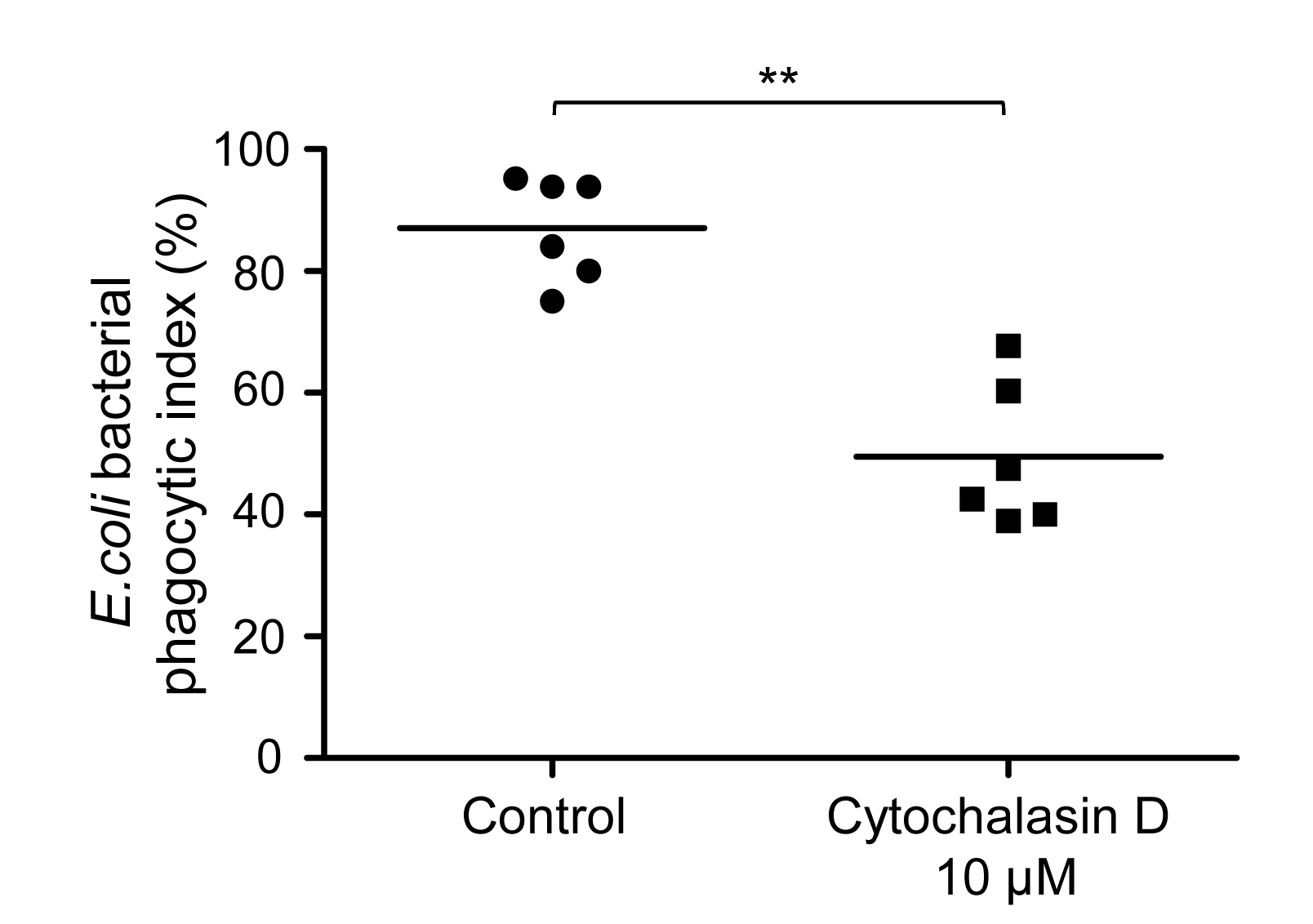

Supplement: Figure S4 — Inhibition of phagocytosis by cytochalasin D in non-CF macrophages. Cells were incubated for 16 h with cytochalasin D (10 µM) before 2 hours incubation with heat-inactivated E. coli-fluorescein (MOI: 100). Results are expressed as percentages as follows: (experimental reading minus negative-control reading / positive-control reading minus negative-control reading) x 100, and are scatter plot with mean of six independent experiments respectively for control and cytochalasin D-treated non-CF macrophages. Mann and Whitney test: ** p<0.01 vs control. (TIF) [file pone.0075667.s004.tif]

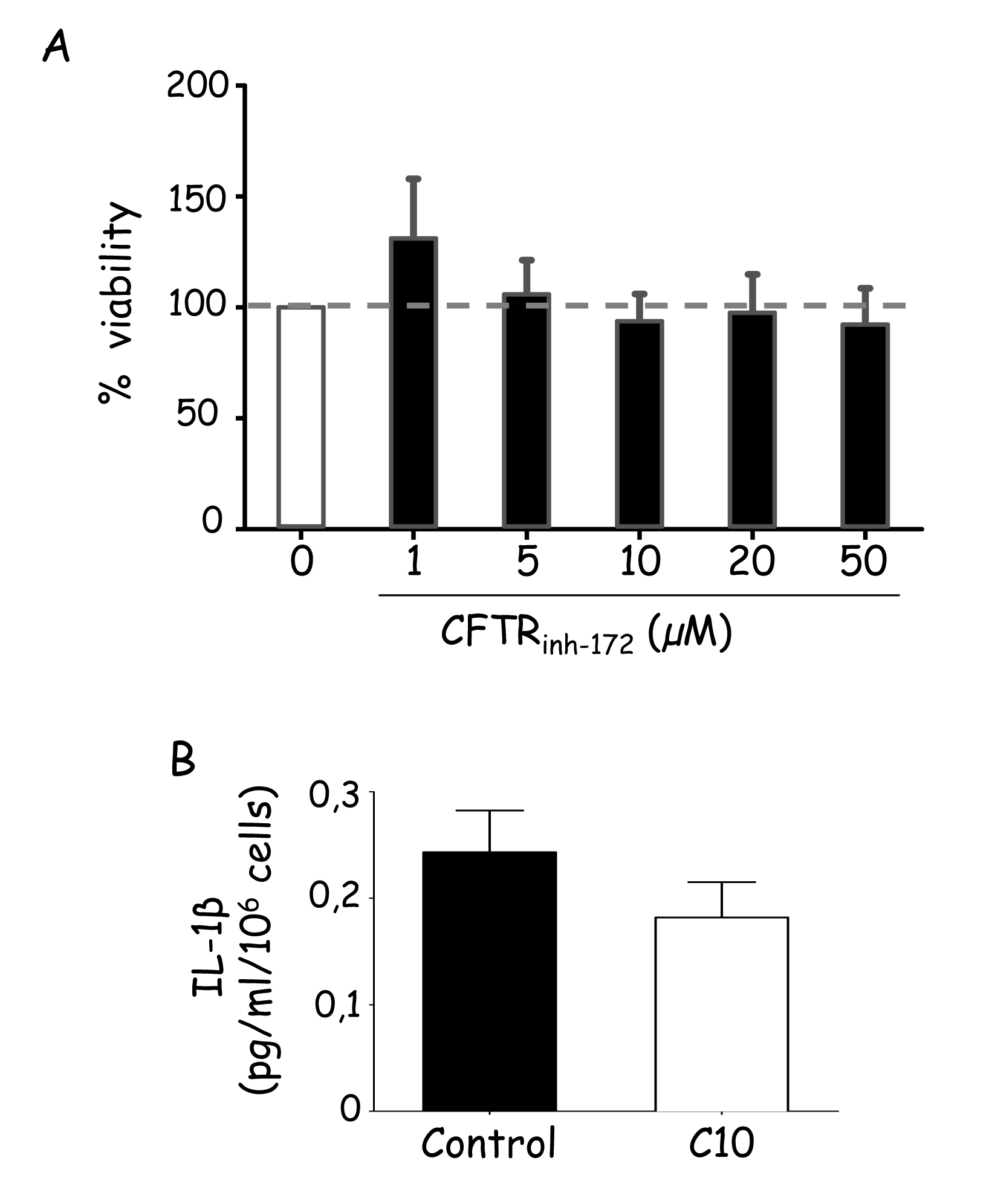

Supplement: Figure S5 — Viability of non-CF macrophages treated by CFTRinh-172 (A). Non-CF macrophages were treated with CFTRinh-172 at 1 to 50 µM (72 h). 100 µl of MTT solution (5 mg/ml in PBS, 3-(4,5-dimethylthiazol-2-yl)-2,5-diphenyltetrazolium bromide, Sigma-Aldrich, Saint-Quentin-Fallavier, France) were added into each well and cells were incubated at 37°C and 5% CO2 for 2 hours. The medium was removed and 100 µl of DMSO was added into each well. The plate was gently rotated on an orbital shaker for 10 min to completely dissolve the precipitation. The absorbance was detected at 540 nm with a microplate reader associated with Genesis software (LabSystems Spectrophotometer, Cambridge, UK). (B) IL-1β levels were measured in supernatants of non-CF macrophages treated or not with CFTRinh-172. IL-1β data are shown as mean ± SEM of eight independent experiments. (TIF) [file pone.0075667.s005.tif]
