# Supplementary material for: Impaired Functions of Macrophage from Cystic Fibrosis Patients: CD11b, TLR-5 Decrease and sCD14, Inflammatory Cytokines Increase
Source: PLoS One. 2013 Sep 30;8(9):e75667. doi: 10.1371/journal.pone.0075667 (PMC3787056; doi:10.1371/journal.pone.0075667)
Supplement: File S1 — Supplemental Information. (DOCX) [file pone.0075667.s006.docx]

**Description of supporting information**

**SUPPORTING INFORMATION METHODS**

**Flow cytometric immunolabelling assay**

Several monoclonal antibodies were used for direct immunofluorescence labeling: PE-conjugated mouse monoclonal antibodies directed against CD71, CD64, CD11b, TLR-1 and CD16; FITC-conjugated mouse monoclonal antibodies directed against CD14, TLR-5, TLR-2 and CD71. All these monoclonal antibodies were purchased from BD Bioscience (Pont de Claix, France).

***E. coli* phagocytosis assay**

Briefly, 10^5^ CF and non-CF macrophages were cultured into 96-multiwell plates and non-CF macrophages were treated with CFTR-inhibitor (CFTR_inh-172_, 10µM, each day during 72h) or vehicle (dimethylsulfoxide DMSO, 0.003%, Sigma-Aldrich). After incubation, medium was removed and cells were exposed to *E. coli* fluorescent bioparticles for 2 hours in incubator (37°C, 5% CO_2_). The phagocytic activity toward *E. coli* was determined by a fluorescence-quenching assay of non-phagocytosed bacteria with trypan blue after removed the bioparticles suspension from all the wells. Phagocytosis was determined by reading the microplate in a fluorescent plate reader (Gemini XPS Fluorescence Microplate Reader, Molecular Devices, Wokingham, Berkshire, UK) using 480 nm excitation and 520 nm emission. Treatment with cytochalasin D (10 µM, 16h, Sigma-Aldrich) served as control for each experiment. Bacterial phagocytic index was calculated as follows: (experimental reading minus negative-control reading/positive-control reading minus negative-control reading) and expressed in percentage (x100).

**RNA isolation and reverse transcription-real time quantitative PCR (RT-qPCR) analysis**

RNA expression was analyzed through RT-qPCR assays. Briefly, total RNA was isolated from cells using TRIzol (InVitrogen, Life Technologies, Saint-Aubin, France). Total RNA (1 μg) was then reverse transcribed using the RT Applied Biosystem kit (Courtaboeuf, France). qPCR assays were next performed using the Power SYBR Green PCR Master kit according to the manufacturer's instructions (Life Technologies) and an ABI Prism 7300 detector (Applied Biosystem). Gene-specific primers for IL-1β and IL-8 were Quantitect®Primer Assay obtained from Qiagen (Courtaboeuf, France) for each gene: Hs_IL1B_1_SG (NM_000576) ; Hs_IL8_1_SG (NM_000584) and Hs_RRN18S_1_SG (X03205). Gene-specific primers for IL-10 (s. CCTGGAGGAGGTGATGCCCCA, a.s. CCTGCTCCACGGCCTTGCTC), IFN-γ (s. TCCAAGTGATGGCTGAACTG, a.s. CTCTTCGACCTCGAAACAGC), IL-6 (s. AGGCACTGGCAGAAAACAAC, a.s. TTTTCACCAGGCAAGTCTCC) were purchased from Sigma (La Verpillière, France) and Eurogentec (Angers, France) for TNF-α (s. AACCTCCTCTCTGCCATC, a.s. ATGTTCGTCCTCACA). Amplification curves of the PCR products were analyzed with the ABI Prism SDS software using the comparative cycle threshold (CT) method. Relative gene expression was calculated by comparing the number of thermal cycles that were necessary to generate threshold amounts of product (CT). CT was calculated for the IL-1β and IL-8 genes and for the housekeeping gene 18s. For each cDNA sample, the 18s CT was subtracted from the CT for the IL-1β or IL‑8 gene to yield ΔCT, thus normalizing the initial amount of RNA used. The amount of mRNA was calculated as 2^–ΔΔCT^, where ΔΔCT is the difference between the ΔCT of the two cDNA samples to be compared.

**SUPPORTING INFORMATION LEGENDS**

**Figure S1. Characterization of human primary macrophages.** Flow cytometric graph for CD71 membrane expression using an anti-CD71-FITC (isotypic control, grey pick) on non-CF monocytes (white pick, solid line) and non-CF (white pick, black dotted line) or CF macrophages (white pick, green dotted line). Results are representative of five, twenty-four and seventeen independent experiments respectively for monocytes, non-CF and CF macrophages (patients 1-9, 11, 13 and 15-20; table S1).

**Figure S2. mRNA expression of inflammatory cytokines in CF *vs* non-CF macrophages under basal conditions.** mRNA levels were determined by RT-qPCR. Data are expressed relatively to mRNA level found in non-CF cells and are shown as mean ± SEM of four independent experiments (patients 23 and 34-36; table S1). Mann and Whitney test: * p< 0.05 *vs* non-CF macrophages.

**Figure S3. Effect of TLRs agonists on TLRs expression (left panel) and IL-1β and TNF-α secretion (right panel) in non-CF CD71^+^ macrophages.** Expression was analyzed by flow cytometry and expressed as mean fluorescence intensity (MFI, arbitrary unit of fluorescence intensity). IL-1β and TNF-α level were assessed by ELISA. Data are shown as mean ± SEM of four independent experiments. Mann and Whitney test: * p<0.01 *vs* control for IL-1β and § p<0.01 *vs* control for TNF-α.

**Figure S4. Inhibition of phagocytosis by cytochalasin D in non-CF macrophages.** Cells were incubated for 16 h with cytochalasin D (10 µM) before 2 hours incubation with heat-inactivated *E. coli*-fluorescein (MOI: 100). Results are expressed as percentages as follows: (experimental reading minus negative-control reading / positive-control reading minus negative-control reading) x 100, and are scatter plot with mean of six independent experiments respectively for control and cytochalasin D-treated non-CF macrophages. Mann and Whitney test: ** p<0.01 *vs* control.

**Figure S5. *(A)*** **Viability of non-CF macrophages treated by CFTR_inh-172_.** Non-CF macrophages were treated with CFTR_inh-172_ at 1 to 50 µM (72 h). 100 µl of MTT solution (5 mg/ml in PBS, 3-(4,5-dimethylthiazol-2-yl)-2,5-diphenyltetrazolium bromide, Sigma-Aldrich, Saint-Quentin-Fallavier, France) were added into each well and cells were incubated at 37°C and 5% CO_2_ for 2 hours. The medium was removed and 100 µl of DMSO was added into each well. The plate was gently rotated on an orbital shaker for 10 min to completely dissolve the precipitation. The absorbance was detected at 540 nm with a microplate reader associated with Genesis software (LabSystems Spectrophotometer, Cambridge, UK). ***(B)* IL-1β levels were measured in supernatants of non-CF macrophages treated or not with CFTR_inh-172_.** IL-1β data are shown as mean ± SEM of eight independent experiments.
