# Supplementary material for: Impaired Functions of Macrophage from Cystic Fibrosis Patients: CD11b, TLR-5 Decrease and sCD14, Inflammatory Cytokines Increase
Source: PLoS One. 2013 Sep 30;8(9):e75667. doi: 10.1371/journal.pone.0075667 (PMC3787056; doi:10.1371/journal.pone.0075667)
Supplement: Table S1 — Characteristics of CF patients. (DOC) [file pone.0075667.s007.doc]

**Table S1:** Characteristics of CF patients

| **Patient** | **Sex** | **Age** | **Genotype** | **Microbiology** | **FEV1**  **% predicted** |
| --- | --- | --- | --- | --- | --- |
| 1 | M | 28 | F508del/5G>A | A.f., S.a. | 46 |
| 2 | F | 19 | F508del/G551D | S.a. | 119 |
| 3 | M | 41 | F508del/G551D | P.a., S.a. | 51 |
| 4 | F | 33 | F508del/E60X | A.f., S.a. | 44 |
| 5 | F | 37 | F508del/W486X | P.a. | 31 |
| 6 | M | 46 | F508del/S1235R | S.a. | 87 |
| 7 | M | 43 | F508del/F508del | P.a. | 26 |
| 8 | F | 52 | F508del/3272-26G>A | S.a. | 81 |
| 9 | F | 42 | F508del/2789+5G>A | A.f., S.a. | 55 |
| 10 | F | 28 | F311L/G551D | S.a. | 77 |
| 11 | M | 28 | F508del/F508del | S.a. | 43 |
| 12 | F | 32 | F508del/F508del | P.a., S.a. | 34 |
| 13 | M | 48 | F508del/2789+5G>A | S.a. | 91 |
| 14 | F | 19 | F508del/F508del | S.a. | 96 |
| 15 | M | 19 | W119X/G551D | A.f., P.a., S.a. | 87 |
| 16 | M | 32 | F508del/F508del | A.f. | 88 |
| 17 | F | 33 | R600S/dupli4-10 | S.a. | 78 |
| 18 | M | 44 | F508del/2789+5G>A | A.f., S.a. | 72 |
| 19 | F | 32 | F508del/N1303K | P.a. | 28 |
| 20 | F | 25 | 1248+1G>A | S.a. | 93 |
| 21 | M | 22 | F508del/F508del | P.a., S.a. | 53 |
| 22 | M | 20 | F508del/F508del | A.f., S.a. | 62 |
| 23 | M | 22 | F508del/F508del | A.f., P.a., S.a. | 79 |
| 24 | M | 35 | F508del/F508del | S.a. | 93 |
| 25 | M | 22 | F508del/F508del | S.a. | 91 |
| 26 | F | 28 | F508del/F508del | P.a., S.a. | 64 |
| 27 | M | 28 | F508del/F508del | A.f., P.a. | 35 |
| 28 | M | 28 | 1248+1G>A | A.f., P.a., S.a. | 103 |
| 29 | M | 34 | F508del/3898insC | A.f., P.a. | 59 |
| 30 | F | 36 | F508del/F508del | P.a. | 47 |
| 31 | F | 30 | F508del/1282WX | A.f., P.a. | 19 |
| 32 | M | 20 | F508del/F508del | A.f., P.a. | 90 |
| 33 | F | 18 | F508del/deltaI507 | S.a. | 78 |
| 34 | M | 32 | F508del/F508del | P.a., S.a. | 82 |
| 35 | M | 36 | F508del/F508del | P.a. | 51 |
| 36 | F | 30 | F508del/1248+1G>A | A.f., P.a. | 33 |
| 37 | M | 29 | F508del/F508del | S.a. | 70 |
| 38 | F | 25 | F508del/G551D | S.a. | 96 |
| 39 | F | 30 | F508del/F508del | A.f., P.a., S.a. | 65 |
| 40 | M | 26 | F508del/F508del | S.a. | 63 |
| 41 | M | 34 | F508del/1078delT | A.f., P.a. | 61 |
| 42 | M | 21 | F508del/F508del | S.a. | 88 |
| 43 | F | 23 | F508del/F508del | A.f., P.a. | 53 |
| 44 | M | 24 | F508del/F508del | P.a., S.a. | 93 |
| 45 | F | 35 | F508del/p.A1025D | A.f. | 91 |
| 46 | M | 28 | F508del/1789insTA | P.a | 29,5 |

**Abbreviations :** A.f.: *Aspergillus fumigatus;* P.a.: *Pseudomonas aeruginosa*; S.a.: Staphylococcus *aureus*

*FEV1: Force Expiratory Volume in one second*
